# Anti-Inflammatory Performance of Lactose-Modified Chitosan and Hyaluronic Acid Mixtures in an In Vitro Macrophage-Mediated Inflammation Osteoarthritis Model

**DOI:** 10.3390/cells9061328

**Published:** 2020-05-26

**Authors:** Elena Tarricone, Elena Mattiuzzo, Elisa Belluzzi, Rossella Elia, Andrea Benetti, Rina Venerando, Vincenzo Vindigni, Pietro Ruggieri, Paola Brun

**Affiliations:** 1 Department of Molecular Medicine, Histology Unit, University of Padova, 35121 Padova, Italy; elenatarricone@gmail.com (E.T.); elena.mattiuzzo@unipd.it (E.M.); ross.elia@libero.it (R.E.); benettiandrea94@gmail.com (A.B.); 2 Musculoskeletal Pathology and Oncology Laboratory, Orthopedic Clinic, Department of Surgery, Oncology and Gastroenterology, University of Padova, 35128 Padova, Italy; elisa.belluzzi@gmail.com; 3 Department of Molecular Medicine, University of Padova, 35121 Padova, Italy; rina.venerando@unipd.it; 4 Clinic of Plastic and Reconstructive Surgery, University of Padova, 35128 Padova, Italy; vincenzo.vindigni@unipd.it; 5 Orthopedic Clinic, Department of Surgery, Oncology and Gastroenterology, University of Padova, 35128 Padova, Italy; pietro.ruggieri@unipd.it

**Keywords:** chondrocyte, osteoarthritis, inflammation, chitosan, hyaluronic acid

## Abstract

The development and progression of osteoarthritis (OA) is associated with macrophage-mediated inflammation that generates a broad spectrum of cytokines and reactive oxygen species (ROS). This study investigates the effects of mid-MW hyaluronic acid (HA) in combination with a lactose-modified chitosan (CTL), on pro-inflammatory molecules and metalloproteinases (MMPs) expression, using an in vitro model of macrophage-mediated inflammation. Methods. To assess chondrocyte response to HA and CTL in the presence of macrophage derived inflammatory mediators, cells were exposed to the conditioned medium (CM) of U937 activated monocytes and changes in cell viability, pro-inflammatory mediators and MMPs expression or ROS generation were analysed. Results. CTL induced changes in chondrocyte viability that are reduced by the presence of HA. The CM of activated U937 monocytes (macrophages) significantly increased gene expression of pro-inflammatory molecules and MMPs and intracellular ROS generation in human chondrocyte cultures. HA, CTL and their combinations counteracted the oxidative damage and restored gene transcription for IL-1β, TNF-α, Gal-1, MMP-3 and MMP-13 to near baseline values. Conclusions. This study suggests that HA-CTL mixture attenuated macrophage-induced inflammation, inhibited MMPs expression and exhibited anti-oxidative effects. This evidence provides an initial step toward the development of an early stage OA therapeutic treatment

## 1. Introduction

Osteoarthritis (OA) is a progressive inflammatory degenerative disease that involves all the components of the joint- cartilage, bone, synovium, meniscus and infrapatellar fat pad and whose radiological signs can be found in more than 70% of people over 50 years of age [1,2]. Although the mechanisms underlying the pathology are not well elucidated, it is known that macrophage-mediated inflammation plays a central role in the structural deterioration of cartilage. The extracellular matrix (ECM) molecules are mainly degraded by matrix metalloproteinases (MMPs) induced by inflammatory mediators [3]. Moreover, the concentration and average molecular weight (MW) of hyaluronic acid (HA), the major components of the cartilage ECM, have been shown to decrease significantly, resulting in a compromised function of joints [1]. 

Macrophage-mediated synovial inflammation is considered one of the main drivers of both OA development and progression [4]. Indeed, an increased number of synovial macrophages in OA has been shown to be responsible for an increased expression of pro-inflammatory cytokines [5]. The two major cytokines released by macrophages and implicated in early osteoarthritis (OA) are interlukin-1β (IL-1β) and tumour necrosis factor-α (TNF-α) that “awaken” chondrocytes from the metabolic state of rest and begin to synthesize, not only pro-inflammatory molecules and reactive oxygen species (ROS), that are added to those produced by inflammatory cells, but also MMPs [2]. Moreover, in vitro and in vivo studies revealed also that galectin-1 (Gal-1) and galectin-3 (Gal-3), molecules that bind specifically to β-galactoside sugars, are over-expressed in OA chondrocytes [6]. In particular, Gal-1 acts as a regulator of inflammatory response in articular cells and tissues, either alone or in combination with Gal-3 and Galectin-8 [7,8]. 

On the basis of this recent knowledge about the mechanisms that lead to the development of articular pathology indicates that the ideal OA treatment needs to combine reduction of inflammation and the “stimulation” of cells to repair the damaged tissues, restoring the correct homeostasis. It is therefore necessary to study new treatments and explore new molecules to be used in OA therapy capable of modulating pro-inflammatory molecule production and also able to guide the cells in the repair process.

Current therapies for OA include non-steroidal anti-inflammatory drugs (NSAIDs), chondro-protectors, corticosteroids and HA supplementation [9,10]. In particular, for intra-articular injections, HA preparations with different molecular MWs are currently used. In fact, low and mid- MW HA preparations seem to increase chondrocyte proliferation and ECM production through cluster differentiation 44 (CD44), the main HA receptor that has a crucial rôle in cell-matrix interaction [11,12,13]. High MW HA compounds have been shown to be more effective on the articular viscoelastic properties. Some authors have recently demonstrated that the combined use of both low MW and high MW HA provide good results in the management of patients suffering from hip OA [14]. However, since HA therapeutic activity is limited by its short biological half-life, crosslinking of the molecule [15] as well as combination with other molecules such as chitosan [16,17] have been tested in order to increase both the short-term HA therapeutic activity and/or viscoelastic properties. Chitosan, a biodegradable and biocompatible compound, was chosen for its structural similarity to glycosaminoglycans and for its chondro-protective and anti-inflammatory properties [18,19]. Moreover, the effects of chitosan mixtures have been studied in both rat and rabbit OA models demonstrating its ability to slow down the progression of OA [20,21] and also to decrease synovial inflammation [21]. A lactose-modified chitosan (CTL), was recently proposed for OA treatment since it is able to interact both with HA and Gal-1 [22,23]. Gal-1 seems to be linked to OA cartilage degeneration, acting as master regulator of ECM enzymes [24]. Several studies have demonstrated, not only an in vitro effect of CTL on chondrocyte growth [25], but also a significantly increased in vivo regeneration of cartilage in rat joint injury [26]. It can be hypothesized that the combination of CTL with HA, could, not only reduce cartilage inflammation promoted by macrophages, but also be able to prolong and support the chondro-protective effect of HA over time, while countering the rôle of Gal-1 in cartilage degeneration. 

The aim of the present study was to test the effect of mid-MW HA in combination with CTL on a macrophage-mediated inflammation in vitro model. For this purpose, we exposed monolayer cultured primary chondrocytes to conditioned medium (CM) of U937 human activated monocytes (macrophages) in the presence of HA and CTL to assess changes in cell viability, in pro-inflammatory genes transcription and in intracellular ROS generation. 

## 2. Materials and Methods

### 2.1. Hyaluronic Acid (HA) and Lactose Modified Chitosan (CTL) Standard Solutions

HA and CTL standard solutions were prepared as follows: sodium HA (250 mg dry basis, MW 1000 to 1600 kDa, obtained from *Streptococcus zooepidemicus*, HTL, Javene, France) was dissolved in 25 mL saline phosphate. CTL (Chitlac® hydrochloride, 250 mg, Jointherapeutics, Como, Italy; chitosan starting material obtained from snow crabs, *Chionoecetes opilio*) were dissolved in 25 mL and then pH adjusted to 7.4. The resulting solutions were transferred into vials and steam sterilised at 121 °C for 15 min. 

### 2.2. Cell and Tissue Culture

For this study human articular chondrocytes isolated from joint cartilage biopsies of OA patients who needed total knee or hip replacement and human U937 cell line were used. 

#### 2.2.1. Human Chondrocytes

After the approval of the Local Ethical Committee (AOP1617) and after obtaining the patients‘ written informed consent, human articular chondrocytes were isolated from joint cartilage biopsies of OA patients with a standard procedure [27]. Briefly, the biopsies were finely chopped and treated with 0.25% trypsin for 15 min (Invitrogen, Carlsbad, CA, USA) and overnight at 37°C with type I collagenase (100 U/mL, Worthington Biochemical, Lakewood, NJ, USA). The digested material was then re-suspended in Dulbecco’s modified Eagle’s medium (DMEM) plus foetal bovine serum (FBS, 10%), 50 μg/mL L-ascorbic acid (Sigma, St Louis, MO, USA), 1% penicillin/streptomycin (P/S), and 1.2% glutamine (all from Gibco, ThermoFisher, Waltham, MA, USA). The cells obtained were expanded, the medium was renewed daily, and the viability of the cells was tested by tryptan blue staining. 

#### 2.2.2. U937 Monocytes and Their Activation to Macrophages

Human monocytes U937 were purchased from Thermo Scientific (Wilmington, NC, USA) and were differentiated in adherent macrophages by treatment with 50 ng/mL phorbol myristate acetate (PMA, Sigma) for 48 h and 1 µg/mL lipopolysaccharides (LPS, Sigma) for 1 h. The differentiation of monocytes to macrophages was examined under an inverted phase-contrast microscope and the mRNA expression of the macrophage differentiation marker CD68 was analysed by quantitative real time PCR (qPCR). Cells were then washed and cultivated with complete RPMI medium for 24 h to produce the inflammatory conditioned medium (CM). The CM was collected, filtered (0.22 μm), stored at −80 °C and used to treat human chondrocyte cultures. 

### 2.3. Evaluation of the Effects of HA and CTL on Human Chondrocyte Viability

To assess the HA and CTL effects on the viability of chondrocytes confluent cells from three different donors were grown in standard culture conditions for different time periods with increasing quantities of the compounds, alone or in combination. In all experiments, chondrocytes were seeded at a density of 7000/cm^2^ in multi-well dishes and viability was determined at designated time intervals (1-3-6 days) by the 3–4,5-dimethylthiazol-2-yl-2,5-diphenyltetrazolium bromide (MTT) test (Sigma) using a modified Denizot method [28]. With this procedure, only viable cells with functioning mitochondria can oxidize MTT to a violet-red reaction product. Each experiment was performed in triplicate, using cell preparations from three different subjects.

### 2.4. Analysis of Anti-Inflammatory and Anti-Oxidative Effects Induced by HA and CTL

The effects of HA and CTL at different concentrations on human chondrocytes exposed to U937 CM for 24h were assessed by analysis of pro-inflammatory molecules expression and by the detection of ROS generation. 

#### 2.4.1. RNA Isolation and Quantitative qPCR Analysis

At 6, 12 and 24 h after treatment, cells were detached and mRNA extracted to analyze the expression of pro-inflammatory cytokines (*IL-1β, TNF-α), Gal-1, MMP-13* and -3, by qPCR. Total RNA was extracted by TRIzol (Life Technologies, Carlsbad, CA, USA) according to the manufacturer’s instruction. RNA quality was controlled using a Nanodrop 2000c spectrophotometer (Thermo Scientific). Subsequently, 500 ng of total RNA was reversely transcribed using oligo-dT and Superscript II (Life Technologies), according to the manufacturer’s instructions. qPCR was performed on a Rotor Gene RG-3000A (QIAGEN, Hilden, Germany) using Xpert fast SYBR (GRISP, Porto, Portugal). Primers used for RQ-PCR analysis are listed in Table 1. Gene expression was evaluated with 2^^-^^ΔΔ^^Ct^ method and all expression values were normalized using expression of peptidylprolylisomerase A (PPIA) as an endogenous control.

#### 2.4.2. Western Blotting Analysis

Human chondrocyte cells were analysed by western blotting for the presence of IL-1β and GAL-1. For this purpose, the cell pellets were treated for 30 min at 4 °C with RIPA lysis buffer (1% *v/v* Triton X-100, 0.5% *w/v* deoxycholic acid, 10 mM EDTA in PBS, Sigma) supplemented with protease inhibitor cocktail. (ThermoFisher, Waltham, MA, USA). After removing particulate material by centrifugation (12,000 g for 10 min, at 4 °C) the supernatants were collected, and protein concentration was determined using the Bradford assay. The lysates (20 μg) were added to the sample loading buffer (62.5 mM Tris pH 6.8, 10% *v/v* glycerol, 2% *w/v* sodium dodecyl sulphate, 5% *v/v* β-mercaptoethanol, and 0.1% *w/v* bromophenol blue), denatured at 98 °C for 5 min and separated by 10% sodium dodecyl sulphate-polyacrylamide gel electrophoresis (SDS-PAGE). Proteins were transferred to Immobilon-P PVDF membrane (Merck-Millipore, Burlington, MA, USA) for 45 min at 4 °C with constant voltage of 100V, in blotting buffer (25 mM Tris, 192 mM glycine and 20% methanol). Non-specific binding sites were blocked incubating PVDF for 1 h at 23 °C in 5% *w/v* non-fat dry milk in 20 mM Tris pH 7.6, 150 mM NaCl (TBS). The PVDF membrane was then incubated with anti-IL-1β (1:1000; MAB601-R&D Systems, Boston, MA, USA), GAL-1 (1:1000; MAB1152-R&D Systems) and β-actin (1:4000; A228, Sigma, St. Louis, MO, USA) antibodies appropriately diluted in TBS, 0.1% Tween-20 (TBST, Sigma) overnight at 4ºC. After three washes for 10 min in TBST, the membrane was incubated with horseradish peroxidase (HRP)-conjugated antibody (anti-mouse IgG, Cell Signaling, Leiden, The Netherlands) diluted in TBST, for 1 h at 23ºC. All membranes were visualized using LiteABlot plus (Euroclone, Pero, Italy), and images were acquired using Image QUANT LAS 4000 imaging system (GE Healthcare, Chicago, Il, USA). Each membrane was stripped with a stripping solution (62.5 mM Tris-HCl pH 6.8, 2% SDS and 100mM β-mercaptoethanol) for 30 min at 50 °C with slight agitation, washed six times for 5 min each in TBST and then reprobed overnight at 4 °C with monoclonal anti-β-actin antibody. Quantitative analysis of western blot was performed by Image J software and the results were normalized to β-actin.

#### 2.4.3. Detection of Intracellular ROS Generation 

At 12 and 24 h human chondrocyte culture treatments with CM, in the presence or absence of HA, CTL or their mixture, cells were trypsin-harvested and the generation of intracellular ROS was measured using 2′,7′-dichlorodihydrofluorescein diacetate (H2DCFDA; Molecular Probes - Invitrogen, Carlsbad, CA, USA), a non-fluorescent probe that is rapidly oxidized to the fluorescent 2′,7′-dichlorofluorescein in the presence of intracellular ROS. Briefly, after washing the cells from trypsin and medium, cells were loaded for 30 min at 37°C with 5 µM H2DCFDA in warm PBS, washed twice and placed in 60 µl of PBS. Fluorescence was measured in a BD FACSCanto™ flow cytometer (Becton, Dickinson and Company, Franklin Lakes, NJ, USA) at excitation and emission wavelengths of 485 and 535 nm, respectively.

### 2.5. Statistical Analysis

Data are reported as the mean and the standard error of the mean (SE). Statistical analyses were performed using the unpaired Student’s t-test and the one-way ANOVA test with multiple comparisons (Fisher LSD test), using GraphPad Prisma 5 (Graph Pad Inc., San Diego, CA, USA) and Stat View Graphics (Abacus Cobcept In., Berkeley, CA, USA). Statistically significant differences are indicated at *p* < 0.05 (*), *p* < 0.01 (**). 

## 3. Results

### 3.1. CTL Induces Changes in Chondrocytes Viability That Are Reduced by the Presence of HA 

**Figure 1:**
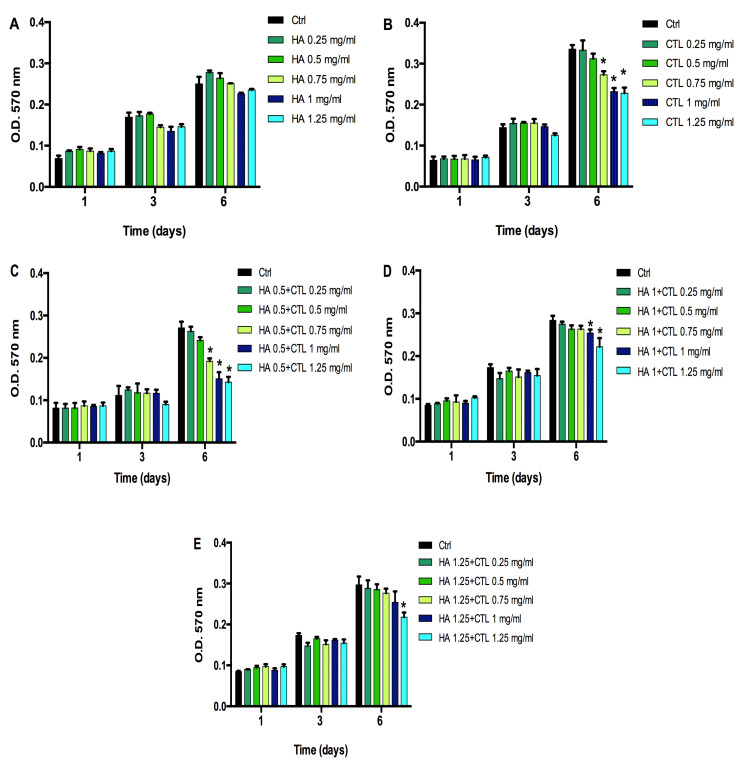
Time-dependent effects of HA and CTL, alone or in combination on human chondrocyte viability. 7000/cm^2^ cells were seeded in 24-well culture dishes and treated at the indicated concentrations of (**A**) HA, (**B**) CTL or different combination of HA-CTL mixture (**C**–**E**). for different exposure times. The MTT test was performed 1, 3 and 6 days after treatment. Data are reported as mean ± SE of three independent experiments. Statistical differences based on unpaired Student’s t-test. * *p* ≤ 0.05 vs. untreated cells.

To determine whether CTL alone or in combination with HA affected the viability of human chondrocytes, cells of three different donors were separately cultured in the presence or in the absence of different concentrations of the molecules (from 0.25 to 1.25 mg/mL). In Figure 1 typical results relative to the cells from one donor were reported. HA, at all the tested concentrations did not affect cell proliferation (Figure 1A). A significant reduction of viability was found in chondrocytes when cells were treated with CTL from 0.5 to 1.25 mg/mL (*p* < 0.05, Figure 1B). However, when CTL was administered to chondrocyte cell cultures in the presence of 0.5, 1 and 1.25 mg HA, (Figure 1C–E), the viability of all treated cells increased significantly and returned to control levels for 0.5 and 0.75 mg/mL CTL in combination with 1 and 1.25 mg/mL HA. In accordance with these findings and with the in vivo results of a previous study [26], most of the subsequent experiments were performed with the mixture of HA 1.25 mg/mL and CTL 0.75 mg/mL.

In accordance with these findings and with the in vivo results of a previous study [26], most of the subsequent experiments were performed with the mixture of HA 1.25 mg/mL and CTL 0.75 mg/mL.

### 3.2. The Conditioned Medium (CM) of Activated U937 Cells Regulated Gene Expression of Human Chondrocytes

**Figure 2:**
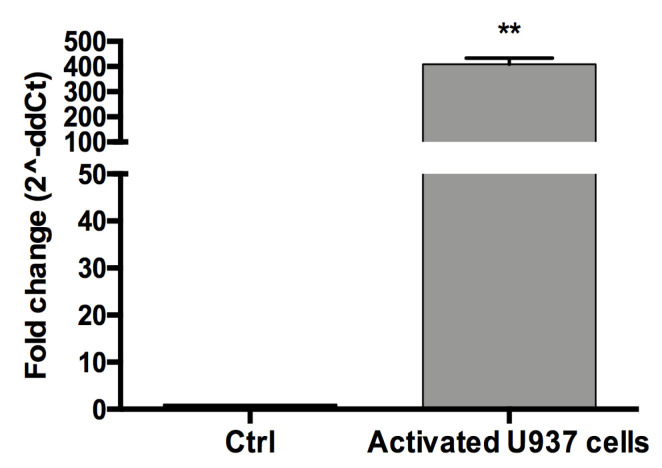
Detection of CD68 in activated U937 human monocytes. Cells were subsequently treated with PMA for 48 h and LPS for 1 h and then mRNA expression of the macrophage differentiation marker CD68 was evaluated by qPCR analysis after 24h. Data are expressed as mean ± SE of three independent experiments. Statistical differences based on unpaired Student’s t-test. ** *p* < 0.01 vs. untreated cells.

**Figure 3:**
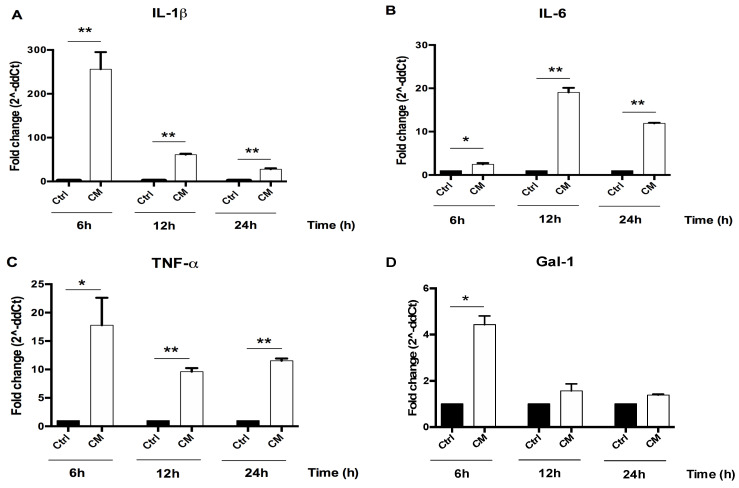
The conditioned medium (CM) of activated U937 cells induced pro-inflammatory molecules expression. Human chondrocytes were exposed for 24h to CM of activated U937 and then cultured under optimal conditions. RNA transcript levels specific for (**A**), *IL-1β*, (**B**) *TNF-α*, (**C**) *IL-6* and (**D**) *Gal-1* were evaluated by qPCR as described in the Materials and Methods section. Statistical differences based on unpaired Student’s t-test. Data are expressed as mean ± SE obtained from at least three independent experiments. CM, chondrocytes cultures exposed to U937 CM. * *p* < 0.05 and ** *p* < 0.01 vs. untreated cells.

PMA and LPS activated U937 human monocytes are able to produce a wide variety of inflammatory molecules released in the culture medium mimicking a natural inflammatory event. The PMA/LPS activation of U937 cells was confirmed by an increased CD68 gene expression (*p* < 0.01, Figure 2) and a significant up-regulation of the pro-inflammatory cytokines *IL-1β TNF-α, IL-6* and *Gal-1* and *-3* (Figure S1). When human chondrocyte cultures were incubated for 24h with the CM derived from the activated U937 cells, a significant increase of *IL-1β, TNF-α*, *IL-6* and *Gal-1* gene expression was detected at 6, 12 and 24 h after treatment (*p* < 0.05) as shown in Figure 3 where the results for the cells of one donor were reported. 

### 3.3. HA, CTL and HA-CTL Mixtures Reduce Chondrocyte Expression of Pro-Inflammatory Molecules

**Figure 4:**
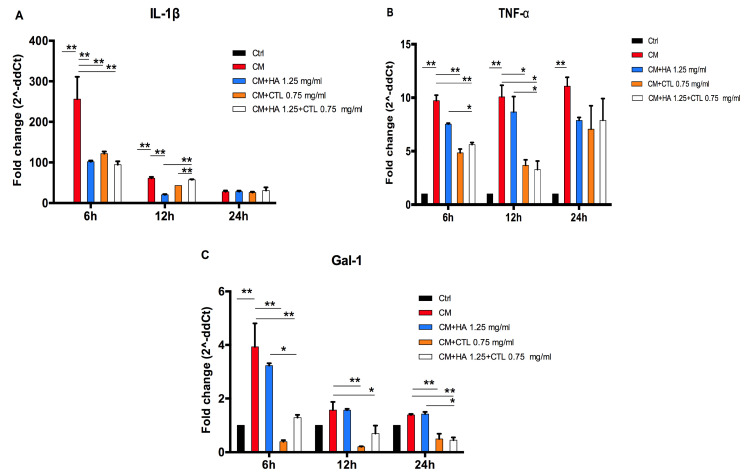
Chondrocyte gene expression of pro-inflammatory molecules after exposure to U937 CM, in the presence or absence of HA, CTL and HA-CTL mixture. Human chondrocytes were exposed for 24h to CM of activated U937 and then cultured in the presence or absence of 1.25 mg/mL HA, 0.75 mg/mL CTL or their mixture. RNA transcript levels specific for (**A**), *IL-1β*, (**B**) *TNF-α* and (**C**) *Gal-1* were evaluated by qPCR as described in the Materials and Methods section at 6, 12 and 24 h after treatment. Statistical differences are based on one-way ANOVA test with multiple comparisons. Data are expressed as mean ± SE obtained from three independent experiments. CM, chondrocytes cultures exposed to U937 CM. * *p* < 0.05 and ** *p* < 0.01 vs. respective CM treated control.

The anti-inflammatory effect of the HA and CTL was tested on chondrocyte cultures treated with the CM of activated U937 human monocytes for 24h. Cell cultures were subsequently treated with the HA, CTL or their mixture, at different concentrations. The results showed that the molecules and their mixture induced a significant down-regulation of *IL-1β* (*p* < 0.01) and *TNF-α* (*p* < 0.05) gene expression at 6 h after treatment and that this effect was still evident at 12 h of culture (Figure 4A,B). At this time point IL-1β expression in the presence of HA-CTL mixture was significantly reduced in comparison with that exerted by single compounds (*p* < 0.05). Moreover, the same mixture leads also to a significant down-regulation of TNF-α expression at 6 and 12h post-treatment, when compared to that induced by HA alone (*p* < 0.05). CTL and HA-CTL mixture, but not HA, significantly decreased the levels of Gal-1 expression (Figure 4C) at 6, 12 and 24 h after treatment (*p* < 0.01).

**Figure 5:**
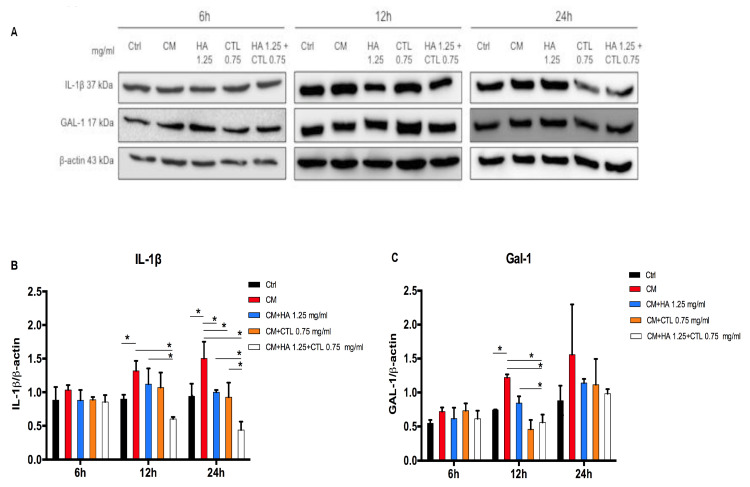
Chondrocyte protein expression of IL-1β and GAL-1 after exposure to U937 CM, in the presence or absence of HA, CTL and HA-CTL mixture. After 24h exposure to CM of activated U937, human chondrocytes were cultured in the presence or absence of 1.25 mg/mL HA, 0.75 mg/mL CTL or their mixture. Protein expressions were analysed by western blotting for IL-1β and Gal-1 at 6, 12 and 24 h after treatment. (**A**): Blotting images of one representative experiment. IL-1β (**B**), Gal-1 (**C**) levels at different time points. Statistical differences are based on one-way ANOVA test with multiple comparisons. Data are expressed as mean ± SE obtained from three independent experiments. CM, chondrocytes cultures exposed to U937 CM. * *p* < 0.05 and ** *p* < 0.01 vs. respective CM treated control.

The western blotting analysis (Figure 5 and Figure S2) confirmed that the CM of activated U937 human monocytes caused an increased expression of IL-1β, and Gal-1. The addition of HA and CTL to inflamed cell cultures induced a down-regulation of IL-1β expression only at 24 h (*p* < 0.05), whereas their mixture at 12 and 24 h (*p* < 0.05, Figure 5B). Furthermore, HA-CTL significantly reduced the expression of IL-1β in comparison with that induced by the single compounds at 12 and 24 h after treatment (*p* < 0.05, Figure 5B). CTL and HA-CTL reduced Gal-1 levels at 12 h after treatment (*p* < 0.05, Figure 5C). 

### 3.4. HA-CTL Mixture Down Regulated the Expression of MMPs

**Figure 6:**
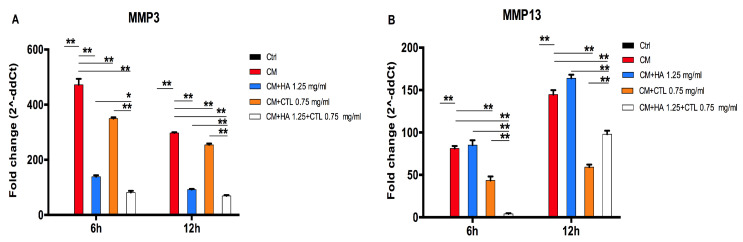
*MMP-3* and *MMP-13* expression of chondrocytes cultures exposed to U937 CM in the presence or absence of HA, CTL and their mixture. Human chondrocytes were exposed for 24h to CM of activated U937 and then cultured in the presence or absence of 1.25 mg/mL HA, 0.75 mg/mL CTL or their mixture. RNA transcript levels specific for (**A**) *MMP-3* and (**B**) *MMP-13* were evaluated by qPCR as described in the Materials and Methods section at 6 and 12h after treatment. Statistical differences are based on one-way ANOVA test with multiple comparisons. Data are expressed as mean ± SE obtained from three independent experiments. CM, chondrocytes cultures exposed to U937 CM. * *p* < 0.05 and ** *p* < 0.01 vs. untreated cells or compared with respective CM treated cells.

Since pro-inflammatory cytokines and Gal-1 may influence the expression of MMPs, the expression of *MMP-13* and *MMP-3* in human chondrocytes treated with the CM of activated macrophages was evaluated (Figure 6). Indeed, it was shown that the exposure of chondrocyte cultures to the CM induces an overexpression of both MMPs. However, HA, CTL and HA-CTL mixture significantly down-regulated *MMP-3* expression at 6 and 12 h after treatment (*p* < 0.01 and *p* < 0.05), whereas only CTL and HA-CTL mixture significantly decreased the levels of *MMP-13* (*p* < 0.01) at both time points. Moreover, MMPs expression, in the presence of HA-CTL mixture was significantly reduced in comparison with that exerted by single compounds (*p* < 0.05 and *p* < 0.001).

### 3.5. HA-CTL Mixture Induced a Reduction of ROS Formation in Human Chondrocytes Exposed to CM of U937 Activated Aonocytes

**Figure 7:**
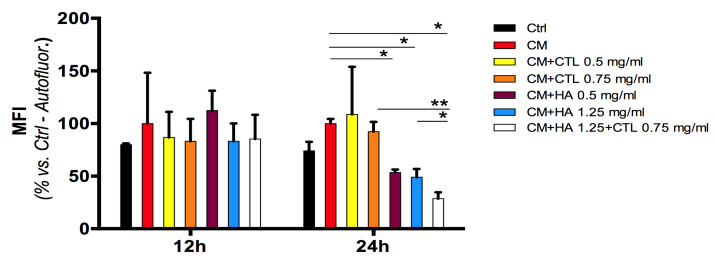
Reactive oxygen species (ROS) generated in human chondrocytes by U937 CM treatment generated in the presence or absence of HA, CTL alone or in combination. Primary cultures of human chondrocytes were exposed to U937 CM for 24 h and then treated for 12 or 24 h with 1.25 mg/mL HA, or 0.75 mg/mL CTL or their mixture. Cells were cultured under optimal conditions and then evaluated at different times for intracellular ROS production using the FACS-activated 2′,7′-dichlorofluorescein diacetate probe, collecting at least 10,000 events. ROS formation is expressed as percentage of U937 CM treated chondrocyte fluorescent intensity. Statistical differences are based on one-way ANOVA test with multiple comparisons (Fisher LSD). Data are reported as mean ± SE of the percentage of fluorescence observed in at least two independent experiments. * *p* < 0.05 compared with respective CM treated control.

Intracellular ROS generation was evaluated using the H2DCFDA probe. As reported in Figure 7 the exposure of human chondrocyte culture to the CM of activated U937 monocytes (macrophages) induced a significant ROS formation when compared with that of untreated control cultures. The subsequent treatment of chondrocyte culture with HA alone or in combination with CTL significantly reduced the production of ROS (Figure 7, *p* < 0.05). In particular, the HA-CTL mixture has an anti-oxidative effect higher than that of the single substances administered individually (*p* < 0.05). 

Throughout these studies we demonstrate not only the anti-inflammatory activity of the mixture HA-CTL but also a consistent link between this activity and anti-oxidative effect of the same compounds. 

## 4. Discussion

The present study demonstrated that the mixture of mid-MW HA and CTL, a lactose-modified chitosan able to bind Gal-1 and HA [23,29], exerted anti-inflammatory and anti-oxidant properties when administered to primary human chondrocyte cultures exposed to the conditioned medium (CM) of activated U9237 monocytes. The reduced inflammation is accompanied by a decrease of matrix proteases expression, responsible for the ECM degradation in early OA. Furthermore, human chondrocytes exposed to CM of activated U937 cells underwent oxidative damage that was less pronounced when these cells were treated with HA and CTL at the moment of in vitro injury, since the mixture was able to increase the HA anti-oxidative properties reported in our previous studies [11]. The correlations between inflammation and oxidative damage is well demonstrated [30] and it was recently reported that the role of HA in scavenging oxygen radicals is correlated to an anti-inflammatory effect through Nrf2 regulation [31].

The presented cell viability data showed that the combination of the two molecules is safe and results in an effective treatment of chondrocytes. In fact, changes in cell viability induced by CTL are reduced by the presence of HA, confirming the well demonstrated protective role of HA in maintaining viability of normal chondrocytes and restoring survival of cells damaged by oxidative stress [32,33].

Macrophages are crucial regulatory cells of host defence, and macrophage-induced inflammation contributes to the disruption of cartilage in the early stage of OA that leads to the imbalance between synthesis and degradation of the various components of cartilage matrix, favouring the degradation process [4,34,35]. U937 activated monocytes are able to produce many pro-inflammatory molecules (unpublished data) that, in turn, induce chondrocyte production of inflammatory activators such IL-1β, TNF-α and galectins. IL-1β and TNF-α are considered key pro-inflammatory mediators in OA, since they induce cartilage degradation and are involved in pain generation [36,37]. Therefore, the inhibition of the IL-1β and TNF-α up-regulation could prevent the degradation of joint cartilage during the pathogenesis of OA. In the present study, we demonstrated that, where chondrocyte culture exposed to macrophage CM, HA and CTL induces the down-regulation, not only of IL-1 β and TNF-α gene expression, but also of Gal-1. Galectins are potent regulators of growth that bind to cell surface glycans with relevance for inflammation [24]. In particular, Gal-1 was recently associated with cartilage degeneration in OA [7,8]. In our study we found that CTL is able to strongly down-regulate Gal-1 expression in comparison to HA and it is reasonable to consider that its effect might be due to the interacting of its lactose residues with Gal-1 which in turn inhibits the interaction of this galectin to the cell surface. This hypothesis is also supported by the findings of Togel et al [38] that reported how lactose reduced the inflammatory effect of Gal-1. In our study we found that the mixture of HA and CTL at the concentration respectively of 1.25 and 0.75 mg/mL decreased transcript levels of IL-1β, TNF-α and of Gal-1 demonstrating the synergistic anti-inflammatory effect of the two molecules. Indeed, the anti-inflammatory properties of HA, a molecule that has a central role in maintaining synovial fluid viscosity, are well documented in clinical studies [39,40]. The differences in cell response to HA depend on its MW, its concentration, or changes in its molecular structure. In particular, mid-MW HA at a concentration of 0.5 mg/mL has been shown to increase normal human chondrocyte proliferation via activation of the CD44 receptor [15]. However, residence time of HA in the joint cavity is short and it was demonstrated that its combination with other molecules such as chitosan [16,17], increased its biological half-life. Indeed, it was recently demonstrated that HA, when administrated together with CTL in a rat OA model [26], it counteracted cartilage degradation even more that HA alone. It could be speculated that the binding of HA chain to CTL creates a polymer network that might prolong the HA therapeutic activity in the joint cavity when compared with native HA. 

The present findings demonstrated also that MMP-3 and MMP-13 expression were up-regulated in chondrocytes culture exposed to the CM of activated U937 cells whereas the HA-CTL mixture not only reduced the pro-inflammatory molecule gene expression but is also accompanied by the decrease of *MMP-3* and *MMP-13* expression. It is well documented that cytokines such as IL-1β, TNF-α and Gal-1 produced by activated synoviocytes or chondrocytes not only suppress the synthesis of ECM [41] but also significantly up-regulate MMP gene expression [42,43]. These up-regulations in early OA are well demonstrated for MMP-3 and MMP-13 [44,45,46] and suggest that MMP gene expression is relevant to OA pathogenesis. Our study demonstrated that HA and CTL are able not only to neutralize the up-regulation of IL-1β, TNF-α and Gal-1, but also to strongly decrease MMP gene expression.

In conclusion, the mixture of HA-CTL, combining its anti-inflammatory and protective effects with the capability of restoring cell viability and the rheological properties of synovial fluid, appears to have a therapeutic potential to inhibit the development of OA and improve cartilage repair. Further in vitro *and* in vivo investigations should be performed to confirm these promising therapeutic applications of HA-CTL mixture. 

## Figures and Tables

**Table 1:** Genes investigated and primers used.

Gene (Accession Number)	Name	Primer Sequences
PPIA (NM_021130.5)	Peptidylprolyl Isomerase A	Fw 5^′^-GGGCTTTAGGCTGTAGGTCAA-3^′^ Rv 5^′^-AACCAAAGCTAGGGAGAGGC-3^′^
IL-1β (NM_000576.3)	Interleukin 1 beta	Fw 5^′^-GAATCTCCGACCACCACTACAG-3^′^ Rv 5^′^-TGATCGTACAGGTGCATCGTG-3^′^
TNF-α (NM_000594.4)	Tumour necrosis factor alpha	Fw 5^′^-AAGCCTGTAGCCCATGTTGT-3^′^ Rv 5^′^-GGACCTGGGAGTAGATGAGGT-3^′^
GAL-1 (NM_002305.4)	Galectin 1	Fw 5^′^-TCTCGGGTGGAGTCTTCTGA-3^′^ Rv 5^′^-GTTCAGCACGAAGCTCTTAGC-3^′^
MMP-3 (J03209.1)	Matrix metalloproteinase 3	Fw 5^′^-TCACTCACAGACCTGACTCG-3^′^ Rv 5^′^-AAAGCAGGATCACAGTTGGC-3^′^
MMP-13 (AY741163.1)	Matrix metalloproteinase 13	Fw 5^′^-AACGCCAGACAAATGTGACC-3^′^ Rv 5^′^-AGGTCATGAGAAGGGTGCTC-3^′^

## Supplementary Materials

The following are available online at https://www.mdpi.com/2073-4409/9/6/1328/s1; Supplementary Figures are added in separate PDF file and listen: Supplementary Figure S1. Pro-inflammatory cytokines expression of activated U937 cells. Human monocytes U937 were differentiated in adherent macrophages by treatment with 50 ng/ml PMA for 48 h and 1 µg/m LPS, Sigma for 1 h. RNA transcript level for (A) IL-1β, (B) TNFα, (C) IL-6, (D) Gal-1, (E) Gal-3 was analysed by qPCR. Data are expressed as mean ± SE obtained from three experiments. * *p* < 0.05 and ** *p* < 0.01 vs. untreated cells; Supplementary Figure S2. Full images of immunoblots for Figure 5B,C.
