# Supplementary material for: Anti-Inflammatory Performance of Lactose-Modified Chitosan and Hyaluronic Acid Mixtures in an In Vitro Macrophage-Mediated Inflammation Osteoarthritis Model
Source: Cells. 2020 May 26;9(6):1328. doi: 10.3390/cells9061328 (PMC7349682; doi:10.3390/cells9061328)
Supplement: Supplementary file 1 [file cells-09-01328-s001.pdf]

Supplementary files

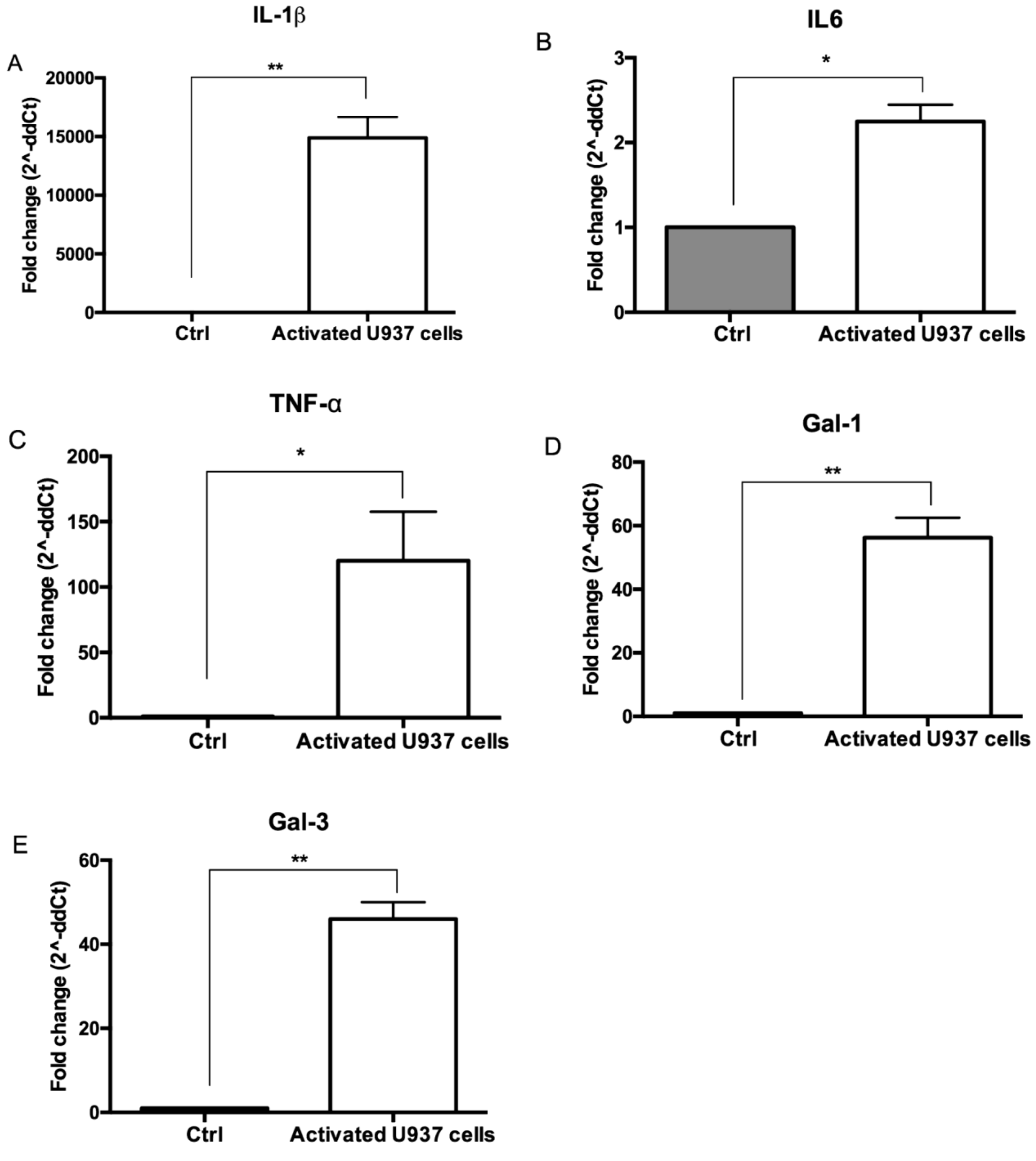

Supplementary Figure S1. **Pro-inflammatory cytokines expression of activated U937 cells**. Human monocytes U937 were differentiated in adherent macrophages by treatment with 50 ng/ml PMA for 48 h and 1 μg/m LPS, Sigma for 1 h. RNA transcript level for A) IL-1β, B) TNFα, C) IL-6, D) Gal-1, E) Gal-3 was analysed by qPCR. Data are expressed as mean ± SE obtained from three experiments. *P<0.05 and **P<0.01 vs. untreated cells.

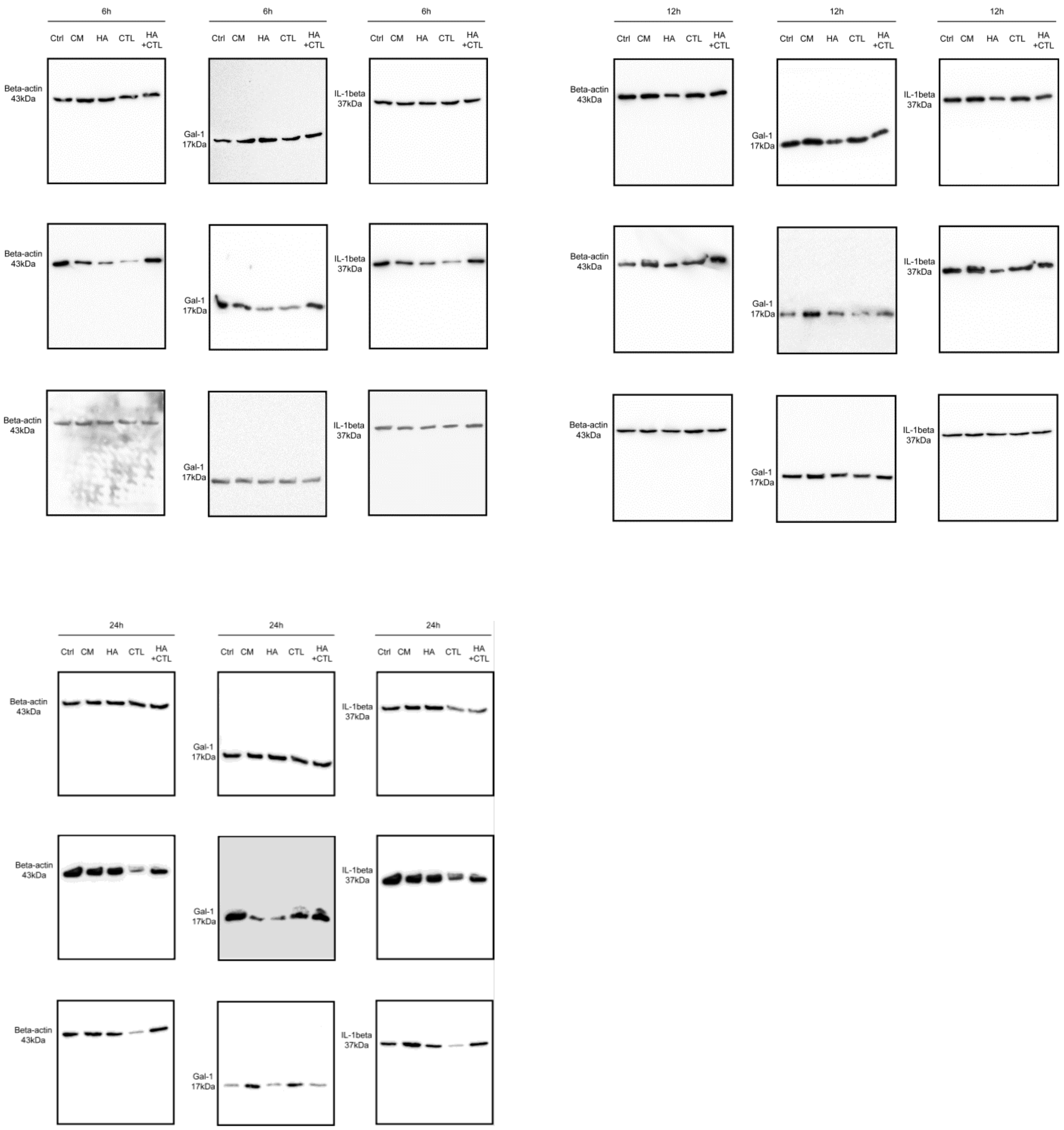

Supplementary Figure S2. **Full images of immunoblots for Figure 5B and Figure 5C**
